# Right ventricle involvement in patients with breast cancer treated with chemotherapy

**DOI:** 10.1186/s40959-024-00224-2

**Published:** 2024-04-15

**Authors:** Ludovico Rossetto, Daniela Di Lisi, Cristina Madaudo, Francesco Paolo Sinagra, Antonio Di Palermo, Oreste Fabio Triolo, Grazia Gambino, Antonella Ortello, Alfredo Ruggero Galassi, Giuseppina Novo

**Affiliations:** 1 Division of Cardiology, University Hospital Paolo Giaccone, Palermo, Italy; 2 Department of Health Promotion, Mother and Child Care, Internal Medicine and Medical Specialties (PROMISE) “G. D’Alessandro”, University of Palermo, Via del Vespro, 129, Palermo, Italy

**Keywords:** Cardiotoxicity, Cardio-oncology, Right ventricle, CTRCD, Three-dimensional echocardiography, Longitudinal strain

## Abstract

**Background:**

Anthracyclines can cause left ventricular (LV) dysfunction. There is little data about right ventricular (RV) damage during chemotherapy.

**Aim:**

This study aimed to investigate the toxic effects of chemotherapy, analyzing its impact on right ventricular function.

**Material and Methods:**

A prospective study was conducted, enrolling 83 female patients (55 ± 11 years old) affected by breast cancer treated with anthracyclines. Cardiological evaluation, HFA risk score assessment and comprehensive echocardiogram, including speckle tracking analysis and 3D analysis, were performed before starting chemotherapy (T0) and at 3 (T1), 6 (T2) and 12 months (T3) after beginning treatment. RV function was assessed with tricuspid annular plane excursion (TAPSE), S’ wave of the tricuspid annulus, fractional area change (FAC), RV global longitudinal strain (RV-GLS), free wall strain (RV-FWLS) and RV 3D ejection fraction (RV-3DEF). Subclinical LV CTRCD was defined as a reduction of GLS > 15% compared to baseline. Subclinical RV cardiotoxicity was defined as the co-presence of a relative decrease of 10% from baseline in RV-3DEF and a relative reduction of 15% from baseline RV-FWLS.

**Results:**

After chemotherapy, we found a significant reduction in 2D-LVEF (*p* =  < 0.001) and 3D-LVEF (*p* =  < 0.001), in LV-GLS and RVLS (*p* =  < 0.001), in FAC and TAPSE, also RV-3DEF reduced significantly (*p* = 0.002). 39% of patients developed LV subclinical CTRCD; 28% of patients developed RV subclinical cardiotoxicity. LV and RV changes occurred concomitantly, and no RV echocardiographic parameters were found to predict the development of LV CTRCD and vice-versa.

**Conclusion:**

After anthracyclines-based chemotherapy, LV and RV subclinical damage occurs, and it can be detected early by speckle-tracking and 3D echocardiography.

## Introduction

Antineoplastic drug-induced cardiotoxicity is a rapidly evolving area of scientific interest due to the increasing number of long-term cancer survivors [1]. Previous studies suggested that more than half of patients treated with anthracyclines develop myocardial dysfunction, and a percentage of them, between 5 and 65%, progress to heart failure, depending on the cumulative dose of the chemotherapy [2]. The occurrence of anthracycline-induced cardiotoxicity [3], even if asymptomatic, negatively impacts the cardiac outcome of cancer patients [4], severely limiting therapeutic opportunities, and has been associated with the worst prognosis. Current studies have demonstrated that the recovery of ventricular systolic function indices and the reduction of adverse cardiac events can be achieved with the early institution of cardio-protective therapy with ACE inhibitors and beta-blockers [5]. These data imply the need to monitor patients undergoing antineoplastic treatment for early detection of cardiotoxicity. Evidence from the scientific literature demonstrated the impact of anthracycline chemotherapy on the LV [6]. Current ESC guidelines on cardio-oncology defined chemotherapy-related cardiac dysfunction (CTRCD) based on left ventricular ejection fraction reduction (LVEF), left ventricular global longitudinal strain reduction (LV-GLS), and biomarkers increase [7]. Few data are available regarding RV involvement in Cardio-Oncology; however, it is well known that there is an essential interdependence between RV and LV due to the presence of a pericardium that contains both chambers and via the sharing of interventricular septum, therefore already in stage B Heart Failure RV function could be impaired and should be investigated [8]. Furthermore, a clear definition of CTRCD based on RV echocardiography parameters doesn’t exist. Our study aimed to investigate the impact of anthracycline treatment on the RV, particularly the usefulness of advanced echocardiography techniques such as 3D Echocardiography and speckle-tracking Echocardiography (STE) in early identification of RV damage [9, 10].

## Methods

A prospective study was conducted enrolling asymptomatic female patients with breast cancer scheduled to receive anthracycline therapy (Fluorouracil-epirubicin-cyclofosfamide protocol) for a duration of 4–6 cycles with a cumulative dose of doxorubicin < 450 mg/m^2^ or other equivalent anthracycline dose. The patients followed up at the Oncology Unit of the P. Giaccone University Hospital of Palermo and were evaluated at the Cardio-Oncology outpatient clinic of the same hospital. The study was conducted according to the guidelines of the Declaration of Helsinki and approved by the Ethics Committee of the University Hospital of Palermo, Italy (protocol number 262/2020). The enrolled population is partially superimposable to that one of a previously published study of our group [11].

The inclusion criteria were age > 18, first breast cancer diagnosis, and good echocardiographic acoustic window. The exclusion criteria were history of previous malignancy, previous chemo and radiotherapy, history of ischemic heart disease, life expectancy judged to be less than one year, LV and RV dysfunctions at baseline, valvular heart disease considered to be at least moderate in degree of severity; inadequate echocardiographic acoustic window. The patients included in the study underwent cardiological examination including collection of clinical history and cardiovascular risk factors, assessment of the risk profile of cardiotoxicity using the HFA-ICOS risk in accordance with the latest ESC guidelines, electrocardiogram, and echocardiogram at baseline (T0) and 3 months (T1), 6 months (T2) and 12 months (T3) after starting chemotherapy. A comprehensive echocardiographic study was performed using a General Electric Vivid E 95 machine according to ASE/EACVI recommendation for chamber quantification, systolic and diastolic function measurement, and valvular and pericardial disease [12]. RV systolic function was evaluated by estimating tricuspid annular plane excursion (TAPSE), tissue Doppler imaging (TDI) S' wave of the tricuspid annulus and fractional area change (FAC). The measurement of the 2D strain by speckle tracking echocardiography (STE) was performed using the Echopac V.202 software (GE). Global longitudinal strain (GLS) was obtained as the mean of all segments analyzed in the 3 acquired projections [13]. A 17-segment evaluation model was used. A GLS value of -21.5 ± 2% was considered normal, with a lower normal range (LLN) limit of -18% [14]. RV longitudinal strain (RVLS) was measured at the free wall (RVFWS) in a focused 4-chamber apical view and at the free wall and septum (Global Longitudinal Strain of the RV, GLS-RV). As a reference, we considered normal values of -24.5 ± 3.8% for RV-GLS and -28.5 ± 4.8% for RVFWS [15]. When feasible, the two-dimensional echocardiographic study dedicated to the right sections was integrated with the acquisition of data for the three-dimensional analysis of the right ventricular function [16]. Through a real-time 3D phased-array transducer (4 V-D), 3 D full volume data set of RV over 1–3 cardiac cycles was acquired. The images were recorded using an apical window, and the transducer position was modified for the best visualization of the tricuspid valve, cardiac apex, and RV outflow tract. 3D RVEF was calculated, with offline analysis, using 4D RV semiautomatic quantification function software (4D-AutoRVQ). After long-axis and short-axis view alignment, anatomic landmarks were manually placed at the level of the tricuspid annulus, RV apex, RV/LV anterior and posterior points, and RV free wall points. The algorithm generated the RV model automatically after placing landmarks and manually correcting contours if necessary.

Chemotherapy related LV dysfunction was defined according to ESC guidelines [7]. Subclinical CTRCD was defined as GLS decline > 15% compared to baseline [17].

No clear data exist so far in the literature regarding the definition of RV subclinical dysfunction [18], so we defined RV subclinical cardiotoxicity as the co-presence of the following conditions: relative reduction of 10% from baseline in RV-3DEF and relative reduction of 15% from baseline in RV free wall strain (RVFWS) in analogy concerning the cut off established for the left ventricle [19].

Patients were distinguished into two groups according to the occurrence or not of subclinical LV CTRCD, and their characteristics were compared to identify possible predictors of the dysfunction. The same comparison was performed between patients who developed or did not have RV dysfunction. According to cardiovascular risk stratification, patients received cardioprotective therapy with beta-blockers and ACE-I/ARB [5].

### Statistical analysis

Categorical variables were expressed as absolute frequency (n°) and percentage (%), and continuous variables were described as mean ± standard deviation (SD) or as median and interquartile range as appropriate. To verify the statistical significance of the observed differences between the four times considered (baseline, three months, six months, and one year), a statistical analysis was conducted that allows for multiple comparisons. Since not all the variables included in the analysis respected the assumption of normality, two types of tests were used: for data following a normal distribution, it was used a repeated measures ANOVA model, while for data following a non-normal distribution, it was used Friedman's nonparametric test. The average increases in the individual data were calculated to evaluate possible statistically significant differences between T0 and T1, T1 and T2, and T2 and T3. The existence of statistical significance was investigated by applying the Student's T-test for paired data for variables that follow a normal distribution and the Wilcoxon test for paired data for variables that do not follow a normal distribution. Receiver operating curve (ROC) analysis was performed to determine the sensitivity and specificity of RV echocardiographic parameters in predicting subclinical chemo-related cardiotoxicity. The Spearman correlation test was used to evaluate the strength and direction of the relationship between changes in right and left ventricular function, evaluating the increase or decrease from baseline to follow-ups in right and left ventricular function (LV3DEF /LVGLS and RV3DEF/RVFWGLS) with the aim of examining whether there was a parallel trend towards dysfunction in both parameters. A two-sided *P*-value < 0.05 was considered statistically significant for single tests, whereas, for multiple testing, the significance level was adjusted using Bonferroni correction. All statistical analyses were performed using R studio software, version 1.4.1103 (2009–2021 RStudio, PBC).

## Results

**Fig. 1:**
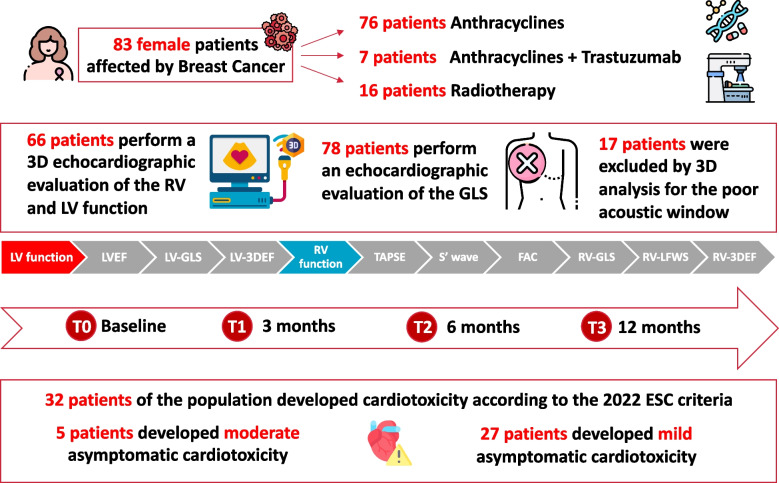
Flow chart of the study

We enrolled 83 female patients affected by breast cancer (mean age 55 ± 11 years). The general characteristics of the study population and the cardiotoxicity risk score assessment using HFA-ICOS score at baseline are reported in Table 1. The most common risk factor was smoking (active or previous, 42%), followed by arterial hypertension (30%). The most used antihypertensive drugs were ACE-inhibitors and beta-blockers. The mean cumulative anthracycline dose was 250 ± 44 mg/m^2^ of doxorubicin. 19% received radiation therapy, and 18% of patients underwent trastuzumab therapy. Most women were at low risk according HFA-ICOS score (65%, *n* = 54). A flow chart of the study is shown in Fig. 1.

**Table 1:** General characteristics of the study population at baseline. Values are reported as mean ± SD and number (n) with percentage (%)

General Characteristics *n* = 83 patients	
Age (years)	55 ± 11
BMI (kg/m2)	26.37 ± 4.35
BSA (m2)	1.65 ± 0.3
Cigarette smoke % (n)	41% (34)
Hypertension % (n)	30% (25)
Family History of CVD % (n)	16% (13)
Dyslipidemia % (n)	11% (9)
Obesity % (n)	10% (8)
Type 2 diabetes % (n)	5% (4)
ACE-I % (n)	10% (8)
Beta-blockers % (n)	10% (8)
Statins % (n)	7% (6)
ARBs % (n)	6% (5)
Diuretics % (n)	6% (5)
Acetylsalicylic acid % (n)	5% (4)
Calcium antagonists % (n)	2% (2)
Radiotherapy % (n)	19% (16)
Trastuzumab % (n)	18% (15)
HFA-ICOS Low Risk % (n)	65% (54)
HFA-ICOS Moderate Risk % (n)	32.5% (27)
HFA-ICOS High Risk % (n)	2.5% (2)

*ACE-I* Angiotensin-converting enzyme *inhibitors*, *ARBs* Angiotensin receptor blockers, *BMI* Body mass index, *BSA* Body surface area

For 66 patients, in addition to using conventional parameters, it was possible to perform an advanced three-dimensional (3D) echocardiographic evaluation of the RV and LV function. The other patients were excluded by 3D analysis for poor acoustic window.

39% (32 patients) of the population developed CTRCD according to the 2022 ESC guidelines [7].

Among the 32 patients who experienced CTRCD, only one patient was treated with ace-inhibitors at baseline, 2 patients were on combination therapy with ARBs and beta-blockers, and 3 patients were treated with beta-blockers alone. Most of the patients who developed CTRCD were not on cardioprotective treatment at baseline (81% vs 19%; *p* < 0.0001). As shown in Table 1, few patients were receiving cardioprotective therapy at baseline so it cannot be stated with certainty that these therapies are statistically protective in our population. CTRCD was more frequent in patients who received radiotherapy compared to those who did not (10/16 pts i.e. 62%, vs 22/67 pts i.e. 33%, *p* = 0.02).

In patients who developed CTRCD, cardioprotective therapy with ACE-I/ARB and or BB was started. Mainly, 6% of the 32 patients who experienced CTRCD developed moderate asymptomatic CTRCD, resulting in a temporary suspension of chemotherapy. In contrast, the others developed mild asymptomatic CTRCD, and none showed the onset of significant symptoms at follow-up. In 38% of cases, left ventricular CTRCD occurred three months after the start of chemotherapy. In 53% of patients 6 months after starting chemotherapy. In 9% of cases, after one year. Echocardiographic data regarding the study population are reported in Table 2.

**Table 2:** Echocardiographic data regarding the study population and differences between timepoints

	T0	*P* value T0 – T1	T1	*P* value T1 – T2	T2	*P* value T2 – T3	T3
LVEDD (mm)	32.3 (3.1)	0.5	32.2 (2.6)	0.13	32.1 (2.7)	0.5	34.4 (8.7)
LV-EDV (ml)	78.7 (11.7)	0.2	81.9 (10.3)	0.1	79.1 (11.5)	0.3	77.8 (11.6)
LV-EF (%)	62.2 (3.5)	0.005	60.8 (3.6)	0.0004	59.2 (4.9)	0.2	58.4 (5.4)
LV-GLS (%)	-20.5 (1.7)	<0.0001	-18.9 (2.1)	0.0012	-17.7 (2.2)	0.15	-18.4 (2)
RV-GLS (%)	-22.2 (2.5)	<0.0001	-20.6 (2.5)	0.0014	-19.6 (2.7)	0.14	-21 (4.1)
RV-FWLS (%)	-26.5 (3.8)	<0.0001	-25.1 (3.8)	0.0001	-23.2 (3.9)	0.3	-24,6 (3.6)
LV-3DEF (%)	63.4 (3.8)	0.008	61.9 (3.4)	0.01	59.9 (4.7)	0.07	57.4 (6.2)
RV-3DEF (%)	59.3 (4.6)	<0.0001	55.1 (4.1)	0.41	54.1 (4.6)	0.2	52.8 (6.1)
TAPSE (mm)	22.4 (2.8)	0.3	22.2 (3.5)	0.02	21.7 (2.4)	0.09	22.3 (2.7)
RV-S’ (cm/sec)	13.5 (2)	0.2	14.3 (2.4)	0.02	13.3 (2)	0.2	11.6 (7.4)
FAC (%)	49.9 (7.7)	0.3	50.9 (4.8)	0.9	50.7 (6.4)	0.001	45.7 (4.7)

*LVGLS* Left ventricle global longitudinal strain, *LVEF* Left ventricle ejection fraction, *LV-3DEF* Left ventricle 3d ejection fraction

**Fig. 2:**
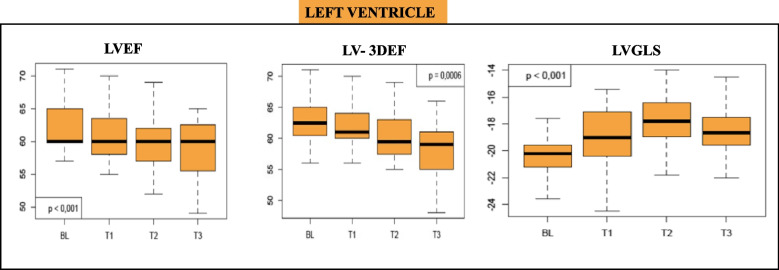
The box plot shows the LV- EF, LV-3DEF, and LV-GLS variations in the study population at T0, T1, T2, and T3 LVEF: Left Ventricle Ejection Fraction; LV-3DEF: Left Ventricle 3D Ejection Fraction; LVGLS: Left Ventricle Global Longitudinal Strain

LV function, assessed by biplane Simpson LVEF measurement and 3D Echocardiography, showed a progressive and statistically significant reduction during monitoring; however, mean LVEF remained above normal limits (see Table 2 and Fig. 2).

Analysis of mean LV-GLS showed a progressive statistically significant decrease at three and six months with stabilization at 12 months (GLS at T0 vs T1 *p* < 0.0001; T1 vs T2 *p* < 0.012; T2 vs T3 *p* 0.15, see Table 2 and Fig. 2).

**Fig. 3:**
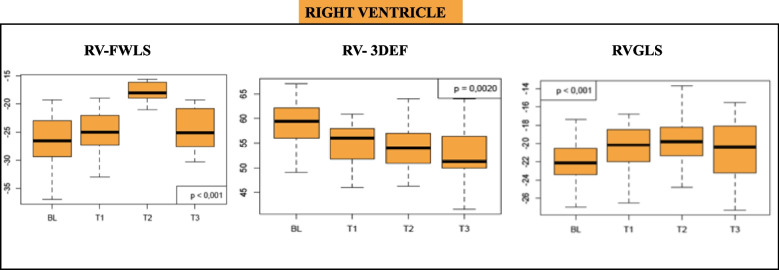
The box plot shows RV-FWLS, RV-3DEF, and RVGLS variations in the study population at T0, T1, T2, and T3. *RV-FWLS: Right Ventricle Free Wall Longitudinal Strain; RV-3DEF: Right Ventricle 3D Ejection Fraction; RV-GLS: Right Ventricle Global Longitudinal Strain*

Considering the RV function: both TAPSE and S’ showed statistically significant variations at T2 [TAPSE T0 vs T1 p 0.3, T1 vs T2 p 0.02, T2 vs T3 p = 0.09;]; [S’ at T0 vs T1 p 0.2, T1 vs T2 p = 0.02; T2 vs T3 p = 0.2]; FAC decrease significantly at T3 (T0 vs T1 p 0.3, T1 vs T2 p 0.9; T2 vs T3 *p* =  < 0.001]; while 3 D and strain parameters changed more precociously [RV-3DEF at T0 vs T1 *p* < 0.0001; T1 vs T2 with *p* 0.041; T2 vs T3 *p* = 0.2], [RV-GLS at T0 vs T1 *p* < 0.0001, T1 vs T2 *p* = 0.0014 T2 vs T3 *p* = 0.14]. See Table 2 and Fig. 3.

**Fig. 4:**
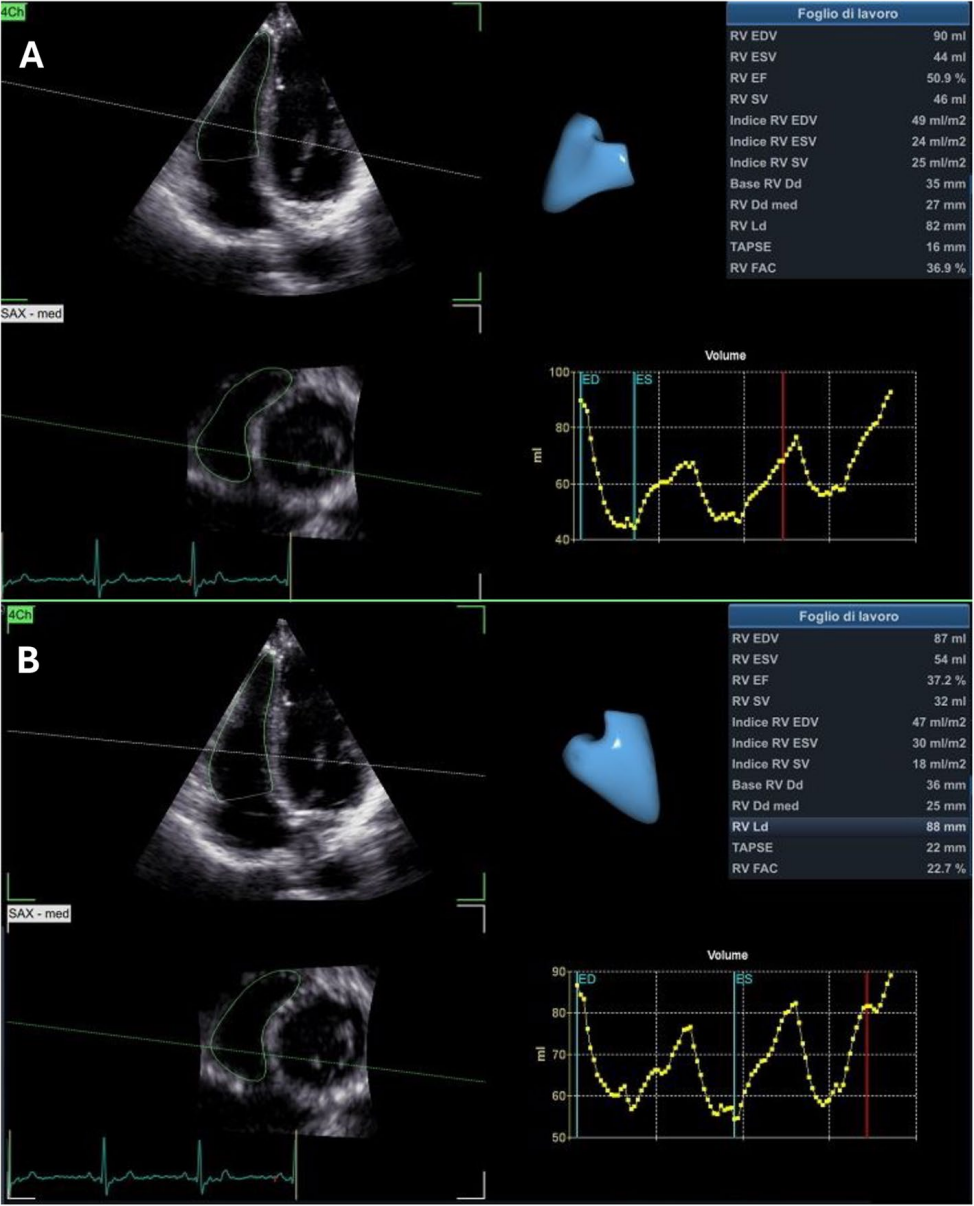
RV-3D echocardiographic evaluation of a patient before (**A**) and after (**B**) chemotherapy treatment

28% (*n* = 8) of patients developed subclinical RV cardiotoxicity, and no patients showed clinical RV cardiotoxicity. In patients who showed a relative reduction in RV-3DEF > 10% from baseline, it was mainly driven by an increase of end-systolic volume (RV-3DESV) [RV-3DESV: 26 ± 5.6 at T0 vs 36.8 mL ± 8.2 at T3, p = 0.0082) rather than end-diastolic volume (3D RV EDV 70 ml ± 11.7 at T0 vs 72.2 ml ± 9.4 at T3, *p* = 0.8964). Figure 4 shows the case of a patient with 3D RV EF reduction after chemotherapy.

All patients with right ventricular dysfunction (*n* = 18) also had subclinical left ventricular dysfunction according to ESC guidelines. So out of 32 patients with CTRCD, 18 also had right ventricular dysfunction (18/32 patients i.e. 56%, vs 14/32 patients i.e. 44%, p = 0.3). RV 3D EF decline correlated with LV 3D EF with a Spearman correlation factor of 0.33 (*p* 0.1); also, RV-FWLS and LV-GLS were related with a Spearman correlation factor of 0.46 (*p* 0.009).

RV subclinical dysfunction did not predict worst LV systolic function parameters at follow-up T3 (EF, LV-3DEF, LV-GLS), as shown in Table 3.

**Table 3:** Comparison between the group with RV dysfunction and the group without RV dysfunction

*T3*	RV dysfunction Group (*n*=18) *Mean (SD)*	No RV dysfunction Group (*n*=48) *Mean (SD)*	*p*-value
LV-GLS (%)	-18.4 (2)	-18.5 (2.2)	0.9304
LV-EF (%)	55 (7.9)	59.6 (3.7)	0.0620
LV-3DEF (%)	54 (7)	59.1 (5.3)	0.0582

*RV-FWLS* Right ventricle free wall longitudinal strain, *RV-GLS* Right ventricle global longitudinal strain, *RV-3DEF* Right ventricle 3d ejection fraction, *TAPSE* Tricuspid annular plane systolic excursion, *RV-S’* Right ventricle s’, *FAC* Fractional area change

Moreover, the characteristics of patients who developed subclinical LV dysfunction were compared with those who did not, and we didn’t find variations between the two groups regarding either the traditional RV systolic function indices (TAPSE, S’TDI, FAC) or the advanced ones (RV-3DEF and RV-GLS) (See Table 4).

**Table 4:** Comparison between the group with LV subclinical dysfunction and the group without LV subclinical dysfunction. Values are reported as mean (SD) and percentage % with number “*n*”

Baseline (T0)	Patients with subclinical dysfunction (relative reduction GLS > 15%) *n* = 27	NO subclinical dysfunction *n* = 51	*p*-value
Age	55 (9.7)	54.7 (10.7)	0.9
Cigarette smoke % (n)	41% (11)	45% (23)	0.7
Hypertension % (n)	41% (11)	27% (14)	0.2
Type 2 diabetes % (n)	7% (2)	4% (2)	0.5
Dyslipidemia % (n)	26% (7)	4% (2)	0.003
Obesity % (n)	11% (3)	10% (5)	0.8
Family History of CVD % (n)	26% (7)	12% (6)	0.1
ACE-I % (n)	19% (5)	6% (3)	0.08
ARBs % (n)	11% (3)	4% (2)	0.2
Beta-blockers % (n)	19% (5)	6% (3)	0.08
Diuretics % (n)	11% (3)	4% (2)	0.2
Calcium antagonists % (n)	7% (2)	0	-
Statins % (n)	15% (4)	4% (2)	0.08
Acetylsalicylic acid % (n)	4% (1)	6% (3)	0.7
RV-FWLS (%)	-27.2 (4.0)	-25.5 (3.3)	0.0430
RV GLS (%)	-22.4 (2.8)	-21.9 (2.1)	0.3577
RV-3DEF (%)	63.2 (3.7)	63.6 (4.0)	0.6567
TAPSE (%)	22.7 (2.7)	22.1 (2.9)	0.2551
RV-S’ (%)	13.6 (1.7)	13.5 (2.4)	0.5695
FAC (%)	50.2 (9.0)	49.6 (4.1)	0.5298

*RV-FWLS* Right ventricle free wall longitudinal strain, *RV-GLS* Right ventricle global longitudinal strain, *RV-3DEF* Right ventricle 3d ejection fraction, *TAPSE* Tricuspid annular plane systolic excursion, *RV-S’* Right ventricle s’, *FAC* Fractional area change

Finally, a further univariate analysis was performed to detect CTRCD predictors (relative reduction of LV-GLS ≥ 15% at follow-up).

Based on the small sample size, none of the cardiovascular risk factors were found to be predictors of subclinical dysfunction. Cigarette smoke sensitivity 99%, specificity 45%, area under the curve (AUC) 0.52 (0.38 – 0.65); arterial hypertension sensitivity 92%, specificity 40%, AUC 0.57 (0.42 – 0.70); diabetes mellitus sensitivity 96%, specificity 47%, AUC 0.52 (0.38 – 0.65); dyslipidemia sensitivity 90%, specificity 54%, AUC 0.70 (0.50 – 0.90); obesity sensitivity 90%, specificity 11%, AUC 0.50 (0.36 – 0.64); family history of cardiovascular diseases sensitivity 88%, specificity 25%, AUC 0.57 (0.43 – 0.70).

However, it couldn’t be identified a value of RV-GLS that can predict the onset of CTRCD (RV GLS T0 sensitivity 99%, specificity 53%, area under the curve 0.66 (0.51 – 0.82); RV GLS T1 sensitivity 99%, specificity 59%, area under the curve 0.78 (0.66 – 0.88); RV-FWLS sensitivity 99%, specificity 53%, area under the curve 0.74 (0.63 – 0.85).

## Discussion

Although the therapeutic protocols used for the treatment of breast cancer have led to an increase in survival, these can cause myocardial damage. Research efforts have focused on evaluating myocardial alterations in an early stage and trying to predict which patients may develop cancer therapy-related cardiac dysfunction (CTRCD) to implement the necessary protective strategies. The guidelines published by the ESC in 2022 recommend the evaluation of LVEF and LV GLS for early detection of CTRCD and a careful baseline evaluation with the HFA/ICOS score developed by the Heart Failure Association (HFA) and the International Society of Cardio-oncology (ICOS) [7, 20]. In clinical practice, few studies have validated this score, which seems to predict CTRCD very well in the most at-risk categories [21]. Furthermore, some new parameters, such as the myocardial work index are becoming increasingly popular in the evaluation of cardio-oncology patients [22, 23]. The performance of the left atrium was also studied to understand whether it could predict left ventricular dysfunction [11, 24].

Ultimately, the scientific literature has focused heavily on the cardiotoxicity of the left ventricle induced by antineoplastic drugs. In contrast, the impact of these drugs on the function of the right ventricle has been poorly studied.

The chemotherapy-induced RV remodelling was shown in an animal model study in which higher fibrosis deposition in the RV-free wall was observed after treatment with anthracycline.

Since no cut-off has been set to identify subclinical cardiotoxicity of the RV chamber, reviewing the literature, we used a definition of 10% relative reduction from baseline values of RV-3DEF [25] and 15% relative reduction from baseline in RV free wall strain (RV-FWLS) [26].

Particularly, Keramida et al., in a small single-centre study, showed that in breast cancer women receiving trastuzumab, independently of previous anthracycline chemotherapy, RV GLS deterioration was predictive of cardiotoxicity. Authors proposed a percent change of − 14.8% compared to baseline as a cut-off to define RV cardiotoxicity with 66.7% sensitivity and 70.8% specificity [26].

Similar results were obtained by El-Sherbeny et al. that demonstrated early changes in RV function by 3D echocardiography and 2D strain analysis in breast cancer receiving anthracycline chemotherapy A cut-off value of RVRFWLS > 19.3% predicted reduction of LV function. [27].

Zhao et al. demonstrated, in 74 patients with diffuse large B-cell lymphoma who received anthracycline-based treatment, that RV strain deterioration was associated with subsequent changes in RVEF [25].

Accordingly our single-center study confirmed the sensitivity of STE to early detect CTRCD, which was observed in 39% of the studied population. In comparison, moderate asymptomatic CTRCD was observed in 6%. No patients developed symptomatic CTRCD.

Advanced echocardiographic techniques were more sensitive than conventional parameters to detect damage. Indeed, STE and 3D Echocardiography revealed a progressive reduction of RV performance earlier than S' TDI, TAPSE and FAC. The damage was not evolutive since we observed stabilization of the 3DEF, RV-GLS and RV-FWLS at 12 months, and no clinically relevant alterations were found.

Comparing patients who developed RV cardiotoxicity vs patients who did not, we didn’t find significant differences concerning LV systolic performance indices, either at baseline or after one year of treatment.

At the same time, no difference was found regarding RV parameters when comparing patients who developed LV subclinical dysfunction at follow-up.

Therefore, RV damage does not seem to predict the occurrence of LV ones; both phenomena show a similar temporal variation, with a maximum and significant decrease six months after the start of chemotherapy and a trend to stabilization at the one-year follow-up. Considering the anatomical and functional ventricular interdependence, these data are not surprising [28]. Accordingly, in our opinion, cardiotoxicity is a global phenomenon that simultaneously and uniformly involves the entire heart and, therefore, both the right and left ventricles and the atria, as we already demonstrated in a previous publication [11].

The significant reduction of RV-STE systolic function indices suggests that as well as for the LV, it’s the subendocardial layer of the RV myocardium, responsible for longitudinal shortening, the most vulnerable to toxic effects of chemotherapy.

Compared with the study of Zhao and Keramida, our study population was different, including breast cancer women, all receiving anthracyclines-based treatment. In addition to Zhao’s study, which is terminated at the end of chemotherapy (after the VI cycle), our study documented the absence of clinical evolution in the early changes of RV function, with stabilization at 12 months and after starting cardioprotection. Moreover, by comparing with other conventional parameters such as TAPSE, S’TDI, and FAC, we documented a greater sensitivity of the RV strain and 3D analysis in detecting subtle RV function changes. Compared to Keramida et al., our study included both strain and 3D analysis, showing similar temporal impairments of both parameters. In conclusion, being previous studies small and monocentric, our results reinforce the poor knowledge of RV involvement during anticancer treatment.

Compared with the study of El-Sherbeny et al., whose method and enrolled population were very similar to ours, we used a different definition of cardiotoxixity, infact we included not only a reduction of ejection fraction > 10% below 50% but also a reduction of GLS > 15% compared to baseline, according to recent ESC guidelines [7], therefore the global rate of CTRCD in our study was higher. Moreover in patients who developed CTRCD, cardioprotective therapy with ACE-I/ARB and or BB was started and we showed that damage was not evolutive since we observed stabilization of the 3DEF, RV-GLS and RV-FWLS at 12 months.

Less evidence exists regarding the prognostic impact of RV damage due to chemotherapy; in a retrospective study that includes 96 patients with diffuse large B-cell lymphoma who received chemotherapy with anthracycline, it was observed that impairment of 3D-RVEF > 12.7% predicts cardiovascular adverse events [29].

In our cohort of patients without significant comorbidities, the occurrence of subclinical RV cardiotoxicity didn’t predict the impairment below the standard limit of conventional echocardiographic parameters of RV function, nor the occurrence of heart failure symptoms at one-year follow-up.

### Limitations of the study

The main limitation of this single centre observational study is the small sample size, further limited by logistical difficulties associated with an outpatient setting during the COVID-19 pandemic. Furthermore, the three-dimensional echocardiographic analysis was performed in a small subset of patients due to inadequate acoustic window at baseline and, above all, after left breast implants.

A further limitation of our study is the absence of biomarkers analysis. Despite surveillance protocols including measurement of biomarkers, the check was carried out by patients in external laboratories, using different assays and therefore, results were not homogeneous to perform analyses. Finally, the absence of a long-term follow-up prevented the evaluation the occurrence of adverse events and consequently to confer a prognostic value to the detected RV abnormalities.

## Conclusion

In patients treated with anthracyclines, RV function, investigated by STE parameters and 3D EF, decreases over time, reaching a peak at 6 months and undergoing stabilization at 12 months.

LV and RV damage occur concomitantly, and no parameters seem to predict the other one. Therefore, cardiotoxicity is a global phenomenon involving uniformly the whole heart.

STE and 3D Echocardiography effectively detect any subclinical RV damage induced by chemotherapy and are more sensitive than conventional parameters. We did not find the occurrence of subclinical ventricular damage to predict the reduction of conventional echocardiographic parameters of RV function below the normal limit or the occurrence of symptomatic CTRCD.

Our results regarding the involvement of the RV after antineoplastic drugs represent a further step forward in the knowledge of cardio-oncology. However, for the limitations mentioned above our results need to be verified by more exhaustive multicentric studies with a longer follow-up.

## Data Availability

All data is available upon request.
